# Emotional States of African Elephants (*Loxodonta africana*) Kept for Animal–Visitor Interactions, as Perceived by People Differing in Age and Knowledge of the Species

**DOI:** 10.3390/ani11030826

**Published:** 2021-03-15

**Authors:** Ilaria Pollastri, Simona Normando, Barbara Contiero, Gregory Vogt, Donatella Gelli, Veronica Sergi, Elena Stagni, Sean Hensman, Elena Mercugliano, Barbara de Mori

**Affiliations:** 1 Department of Comparative Biomedicine and Food Science, University of Padua, viale dell’Università 16, Agripolis, 35020 Legnaro, Italy; 2 Ethics Laboratory for Veterinary Medicine, Conservation, and Animal Welfare, University of Padua, viale dell’Università 16, Agripolis, 35020 Legnaro, Italy; greg@conservationguardians.co.za (G.V.); elena.mercugliano@gmail.com (E.M.); 3 Department of Animal Medicine, Production and Health, University of Padua, viale dell’Università 16, Agripolis, 35020 Legnaro, Italy; barbara.contiero@unipd.it (B.C.); donatella.gelli@unipd.it (D.G.); 4 Conservation Guardians, Shongweni Nature Reserve, Outer West, Kwa Zulu Natal 3610, South Africa; 5 School of Agricultural Sciences and Veterinary Medicine, University of Padua, viale dell’Università 16, Agripolis, 35020 Legnaro, Italy; veronica.s92@hotmail.it; 6 Independent Researcher, Via Ranzani 17, 40127 Bologna, Italy; estagni.res@gmail.com; 7 Adventures with Elephants, Bela Bela, Limpopo 0480, South Africa; info@efaf.co.za

**Keywords:** African elephant, qualitative behavior assessment, free-choice profiling, semi-captive management, welfare, human–animal interaction

## Abstract

**Simple Summary:**

We investigated the use of the qualitative behavior assessment (QBA) to evaluate the emotional state of African elephants managed in captive and semi-captive environments by three groups of people with differing ages and levels of knowledge of the species. We also examined whether their assessments correlated with behaviors exhibited by the animals. Fifteen video-clips of a total of 18 African elephants recorded in three different situations (release from the night *boma*; interactions with visitors; return to the night *boma*) were used. The result of the performed analysis supported that the consensus found was not due to chance. This notwithstanding, all the adjectives used by the three observer groups were not strong descriptors of the consensus variables resulting from statistical analysis. All three groups showed a degree of separation between captive and semi-captive management, with semi-captive animals rated as being in a more positive emotional state. For all three groups of observers, stereotypic “trunk swirling” behavior correlated with negative emotional descriptors. Although definitive evaluation of animal welfare requires the services of experts, more studies are needed to investigate the perception of elephants’ emotional states amongst visitors of different ages and background.

**Abstract:**

This study aimed to investigate how three groups of people of differing ages, and with differing knowledge of the species, perceived the emotional state of African elephants (*Loxodonta africana*) managed in captive and semi-captive environments. Fifteen video-clips of 18 elephants, observed during three different daily routines (release from and return to the night *boma*; interactions with visitors), were used for a free choice profiling assessment (FCP) and then analyzed with quantitative methods. A general Procrustes analysis identified two main descriptive dimensions of elephant behavioral expression explaining 27% and 19% of the variability in the children group, 19% and 23.7% in adults, and 21.8% and 17% in the expert group. All the descriptors the observers came up with showed a low level of correlation on the identified dimensions. All three observers’ groups showed a degree of separation between captive and semi-captive management. Spearman analyses showed that stereotypic “trunk swirling” behavior correlated negatively with first dimension (free/friendly versus sad/bored) in the children’s group; second dimension (agitated/confident versus angry/bored) amongst the adults; and first dimension (active/excited versus agitated/bored) amongst the experts. More studies are needed to investigate other potential differences in assessing elephants’ emotional states by visitors of different ages and backgrounds.

## 1. Introduction

African elephants have complex cognitive abilities, a sophisticated social structure, and a vast behavioral repertoire, and they generally attract a large number of visitors in zoos and other animal facilities [1,2]. Concerns have recently been raised regarding the effects on welfare of conditions under which elephants are held and managed in European and American zoos [3,4]. For instance, a high prevalence of factors which might be indicators that the welfare of the animals is not optimal have been reported. These include stereotypic behaviors [5] and health issues, such as ovarian acyclicity [6,7], obesity, foot problems [8], infectious diseases [9,10], and compromised survivorship [11]. Therefore, ensuring the welfare of elephants in zoos can be challenging [12,13]. For these reasons, there is growing scientific interest in developing, validating, and publishing methodologies for assessing the welfare of elephants in zoos, whose results, include indices to assess behavioral and/or psychological changes and their consequences [12,14,15,16]. However, zoo elephants are not the only elephant populations living in controlled conditions, and the external validity of protocols validated for elephants in zoos has never been investigated [17], leaving open to debate their suitability to be applied to other sub-populations of elephants under human control (e.g., semi-captive elephants in South African and Asian facilities).

In Africa, for instance, the management of elephants housed in game reserves, or in privately-owned facilities usually differs from the management of elephants held in zoos [18]. While elephants in zoos are held in fenced enclosures, elephants held in game reserves and other privately-owned facilities are generally afforded the opportunity to spend part of the day moving freely in the bush. Despite remaining under the supervision of their handlers, the latter are left free to express their natural behavioral repertoire without being subject to the imposition of specific activities, except for safety reasons [19], or in cases where they disperse excessively. Therefore, visitors to most of these facilities are able to observe the animals in their semi-natural environment. Additionally, most South African facilities offer visitors some form of organized animal–visitor interaction. These activities can vary greatly in their style and content including, for example, hearing talks on characteristics of the species, observing elephants in their night areas, or witnessing *ad hoc* training sessions. In some cases, visitors are afforded opportunities to approach the elephants and thus become involved in close-up animal–visitor interactions (AVIs) such as feeding or elephant-back safaris.

In 2013, a group of researchers from the University of Padua began a project aimed at developing a protocol for assessing the welfare of African elephants kept in semi-captive environments, and involved in AVIs in South Africa [17]. This assessment protocol involves a merger between scientific and ethical approaches. In this project, qualitative behavioral assessment (QBA), carried out by experts, was included as a method to validate the emotional valences (negative or positive) both of possible behavioral welfare indicators and of management procedures that could possibly affect welfare (see [17] for details).

Developed by Wemelsfelder and colleagues [20,21], the QBA is a reliable method, and has been cross-validated against quantitative behavioral or physiological measures (for example [22,23]). QBA is empirically based on observation of behavioral signs that reflect an animal’s emotional state and does not consist of unfounded projections of human emotions [20,21,24]. Moreover, as stated by Wemeslfelder and colleagues [20] (p.208), “[...] its being based on human perception does not make it a study of human perception. Human observers and their perceptive powers are used as an assessment tool [...]”. Therefore, it has been shown to represent an integrative evaluation tool for use in animal welfare studies, and is hence included in several protocols for welfare assessment, such as Welfare Quality^®^ and the European Animal Welfare Indicators Project (AWIN) [25,26]. It determines the animal’s physical and physiological state, and additionally represents a valid measure of animals’ demeanor [22,27,28]. The ‘holistic’ approach recognizes animals as sentient beings with different personalities and capable of experiencing positive and negative emotions [29]. QBA uses the ‘whole-animal’ approach, which measures how animals respond to the environment and how they deal with it, rather than measuring only the animal’s physical behavior [30]. Specifically, it focuses on the dynamic expressivity of the behavioral demeanor, characterizing and quantifying it, through the use of lists of descriptors. Such list can either be supplied predefined (e.g., [31,32,33]), or developed through a methodology known as free-choice profiling (FCP), in which each observer generates their descriptors [20,21,28,34].

The QBA has been shown to be able to assess an animal’s affective state quickly, reliably, and non-invasively [32], both under semi-captive and on-farm conditions [31]. Therefore, in the few last years, the QBA has been applied to a range of different species and different contexts [20,27,28,30,32,35,36,37,38]. Recently, it has also been used to address human–animal relationships (HAR) in zoos [34].

The main aim of this study was to investigate how three distinct groups of people differing in age and knowledge of the species (i.e., children, non-expert adults, and experts) perceived the behavior of African elephants in its emotional connotations, using free choice profiling assessment (FCP). Additionally, it aimed at investigating whether a difference exists between the descriptors generated by experts observing elephants held in captive management and elephants held in semi-captive management, and whether similar differences could be found amongst other observers. It is important to note that QBA adjectives proposed for elephants were developed in zoo conditions [14], and the findings demonstrate a fair degree of separation in the experts’ scatterplots between videos recorded in captive and semi-captive management systems. This study, therefore, cautions against using adjectives generated exclusively in a zoological context when developing a QBA for elephants in semi-captive conditions. As a sideline of investigation, we also evaluated whether correlations existed between quantitative behavioral assessment and the results obtained from FCP carried out by experts on the same videos, and whether similar results could be found also when non expert observers’ FCP were concerned. Besides, in the context of the project, the FCP procedures running with children and adults provide an idea of the emotional impact on visitors of the “elephants’ experience”, and the results are therefore potentially useful in ethical evaluations when considering the interests of visitors [17].

## 2. Animals, Materials, and Methods

The research was conducted in Italy and South Africa. Five institutions were chosen, and each facility gave permission to film the animals and collect data. The study was observational in nature and was made in accordance with both the ethical requirements of the participating facilities, and relevant national and international regulations. The husbandry routines of the animals involved were not changed or affected by the study. All the observers gave their consent for inclusion before they participated, and the informed consent of parents of each child was given. When the study was performed, no approval of ethical committees was needed in the country leading the project.

### 2.1. Places, Animals, and Their Management

Five institutions and an overall of 18 African elephants were included in the study: (1) Zoo Safari Ravenna, Italy (44°19′36.4″ N 12°16′29.8″ E) a zoological garden housing two female elephants; (2) Indalu Game Reserve, Mossel Bay, Garden Route, Western Cape Province, South Africa (34°10′56.9″ S 21°48′22.4″ E), where at the time of the study, six elephants were housed, three males and three females; (3) Garden Route Game Lodge, Garden Route, Western Cape Province, South Africa (34°12′31.2″ S 21°38′00.1″ E), which held two male elephants; (4) Adventures with Elephants, Bela Bela, Limpopo Province (24°46′54.1″ S 27°57′05.3″ E) which held seven elephants in the reserve, although only five adult animals were included in the study; (5) National Zoological Garden (NZG), Pretoria, Gauteng Province (25°44′05.1″ S 28°11′24.9″ E) which owned three elephants, a male and two females. The five institutions were chosen for their different management typologies. We considered “captive” facilities to be those in which animals were kept in fenced enclosures for the whole day, and in which food supply was completely provided by human intervention. On the other hand, we defined “semi-captivity” as the condition in which animals were allowed to roam in the so-called “free choice activity” for at least part of the day, thus experiencing a varied environment, rich in vegetation and stimuli, while foraging and socially interacting with their conspecifics [19]. In most of these facilities, during “free choice activity”, the elephants are herded to an area where they are free to choose what to do and to roam around in the bush, without any imposed activity and under the supervision of the handlers. Handlers refrain from interacting with elephants unless for safety and security reasons or to avoid excessive dispersion which could hamper herding at the end of the free choice activity time. All the male elephants included in the study were treated with GnRH (gonadotropin releasing hormone), a non-surgical method of managing testosterone and musth in bull elephants [39] that have reached their sexual maturity. Table 1 provides a summary of the elephants included in the study, the age range of the individuals, whether the animals were trained, whether they were involved in interactions with visitors, and management’s typology.

### 2.2. Videos Gathering and Processing

Videos were collected in Italy and South Africa during a period ranging from October to December 2016. Sessions from Italy were video recorded with a Samsung NX1000 mirrorless camera or a SONY A7R II mirrorless camera. Videos recorded in South Africa were shot with a JVC Everio full HD water-resistant camera. Except in the National Zoological Gardens, video recording took place at different times of day, usually beginning with a first recording session when the animals were released in the morning (usually at 7:00 a.m.) and ending with a final session when the animals went back into their *bomas* or indoor areas for the night (usually at 5:00 p.m.). In the National Zoological Garden, video-recording sessions began around 8:30 a.m., and ended around 7 p.m. The order in which individual animals were filmed was scheduled to change every day. However, because of contingent events (operator safety, environmental and weather conditions, hidden animals, etc.), it was not always possible to comply with the order as planned.

For the present study, fifteen videos were chosen from 1200 collected. In the chosen 15 videos, a total of 18 elephants are visible, for an overall duration of 20:51 min. Each video was cut in post-production.

Of the fifteen videos chosen for this study:

- Five videos show “re-entry”: the elephants in the process of returning to the night *boma* or indoor area Four of these videos show the entire group of animals at their respective facilities, while one video, shot at the National Zoological Gardens, shows only Thandi (the dominant female in her group) since the animals at the NZG were returned separately to the night area. These “re-entry” videos range in length from 1:04 to 2:02 min, for a total duration of 7:29 min.
- Five videos show “release”: the elephants being released from their night areas. These videos are divided as above, and have a duration ranging from 0:51 to 2:01 min, for an overall duration of 6:37 min.
- Five videos show “interaction with humans”. In these videos, the dominant elephant of each group is shown interacting with humans. These videos have a duration ranging from 1:00 to 1:45 min, for an overall duration of 6:45 min.

The decision to film at given times (during entry and release from the night *bomas*, and during animal-visitor interactions) was made for three related reasons: (a) because they were the most similar moments in the elephants’ daily routines in all facilities involved in the study; (b) because it was logistically feasible to video record the animals during these times; and (c) because the re-entry and release times presented opportunities during which it was most likely that (almost) all the elephants in each different facility would be visible in the video recordings. 

### 2.3. Qualitative Behavioural Assessment

#### 2.3.1. Observer Groups

Three mixed-sex groups of Italian observers were involved in the study: one group comprising fourteen children between 8 and 13 years old, one group comprising thirteen adults ranging from 20 to 50 years old, and one group of ten experts in African elephants whose members ranged from 25 to 53 years old. None of the observers in the first two groups had had previous experience of animal observation, qualitative behavioral assessment, free choice profiling methodology, or African elephants. In contrast, the expert group included Italian zookeepers with knowledge of the management of African elephants, veterinarians who work in zoos where African elephants are kept, and more generally, Italian researchers who work with African elephants and are involved in handling elephants, welfare assessment, and animal behavior.

The number of observers in each group, and the number of videos analyzed both fall within the limits of what has already been done in scientific studies using FCP [22,24,28,31,32,33,34,38,40,41].

#### 2.3.2. Free Choice Profiling

In this study, the FCP methodology, as described by Wemelsfelder and colleagues [21], is used to evaluate the behavioral expression of the elephant.

Consistent with normal practice for FCP studies, the FCP methodology employed consisted of two separate phases held on separate dates [21]. In the first phase, observers watched each video. At the end of each video, observers had three minutes to write down terms that in their opinion best described the emotions they thought the animals were feeling. All participants generated terms in their native language (i.e., Italian). In the second phase (on the second date), the same observers were asked to watch the same videos again, in the same sequence as before. Each of the observer’s terms was printed in a list, with each observer receiving a list of their own individual terms. Each term was paired with a visual analogue scale (VAS). Observers were now asked to mark a line on the scale at the point considered appropriate between “minimum” (0 mm) and “maximum” (125 mm), thus determining a quantitative value for each term. If an adjective was deemed irrelevant for a certain video or if the observer considered that the video did not provide adequate information to decide how to rate that adjective, the observer was instructed to tick the space marked “non-pertinent” (NP) that appeared alongside the VAS. To avoid every possible impact, the VAS was left entirely blank with no marks or measurement indicators.

Observers were asked to come up with descriptors completely spontaneously in the first phase, and were also free either to choose new descriptors for each video, or to use terms they had already used for other videos. They were also asked to concentrate on choosing the best possible descriptors, and to refrain from communicating with each other in order to minimize external influences. 

Each observer’s scores were thus obtained by measuring the distance in millimeters between the “minimum” point of the VAS, and the line the observer had drawn on the scale. The scores were entered into 37 data matrices (one for each observer), providing scores for the 18 animals on the basis of the observers’ personal vocabularies. Zeros were added to individual matrices every time the observers left a blank so that all observer configurations acquired equal dimensionality.

### 2.4. General Method of Analysis

Data were analyzed separately for the three observer groups using generalized Procrustes analysis (GPA) through a GenStat software edition written by Françoise Wemelsfelder (Genstat 2016, VSN International, Hemel Hempstead, Hertfordshire, UK). GPA is a multivariate technique that identifies patterns in data that do not consist of fixed variables. It detects the level of consensus between observer scoring patterns, giving the percentage of variation between observer configurations explained by the consensus profile. For more detailed explanation of GPA procedures, see [20]. The statistical significance of this consensus is determined through a permutation or randomization test [42], that allows discriminating whether the consensus is a significant feature of the data set or an artefact of the Procrustean calculation procedures. A one-way Student’s *t*-test (*n* = 100) is used to determine whether the true observer consensus profile falls significantly outside the distribution of randomized profiles (*p* < 0.001). 

GPA provides a Procrustes statistic for each pair of transformed observer configurations, which quantifies the percentage of the total variance between observer configurations explained by the consensus profile. The relative distance between transformed observer configurations and the “best of fit” can be projected visually in a so-called “observer plot”. Principal coordinate analysis (PCO) estimates the center of distributions of the relative distance between the observer and a standard deviation and draws a 95% confidence region for the consensus profile.

Observers lying outside this region are potentially outliers who, in some sense, may differ from other observers in their assessment of samples [20]. Once these outliers are excluded, GPA can be repeated to assess whether and how their data influenced the consent profile. At this stage, observer 12 of the children group was an outlier. Since there was a valid reason (e.g., problems with vocabulary), he was excluded from further analysis. Moreover, observers 4, 5, and 13 of the adult group and observers 1 and 6 of the expert group were outliers and were therefore excluded. The consensus profile of the three groups improved after the first exclusion of outliers. Although other outlier observers were identified in the three groups, it was decided to carry out the outlier exclusion procedure only once because further applications of this process would have reduced the sample size of the three groups too much. 

GPA thus transformed the now 13 different video-scoring (representing elephants-scoring) configurations of the children’s group, the 10 different video-scoring configurations of the adult’s group, and the 8 different video-scoring configurations of the experts’ group into a three multidimensional consensus profile, entirely independent from any interpretation by the experimenter.

Through the principal component analysis (PCA), the number of dimensions of the consensus profile is reduced, identifying the principal axes and determining how much variation between the videos these dimensions explain. Each video was attributed a score on each of these dimensions. Scores were then reflected in several two-dimensional ‘elephant-plots’ showing the distribution of the videos along the principal axes of the consensus profile. A standard error ellipse indicates the reliability for each video’s position on the axes.

These dimensions are then interpreted by correlating them to the original individual observer data matrices. This step of the analysis produces two-dimensional individual observer interpretative ‘word charts’, showing the association between all terms of a particular observer and the two or more principal axes of the consensus profile. The higher a term correlates with an axis (or dimension), the more weight it has as a descriptor for that axis. The extent to which individual observers concur in their judgment of elephants’ expressions is indicated by the degree of semantic convergence between charts. If observer assessments show significant convergence, then the consensus profile can be used to appraise qualitative differences between individual animals, defined by the position of individual animals on the plot.

Given that, in the majority of the studies using FCP, the respondents were native English speakers or generated terms in English, or the terms given in a language other than English were translated into English for statistical analysis (e.g., [40]), the process described above was followed a second time using the English translations of the Italian terms given by respondents instead of using the Italian terms themselves. The original Italian terms were translated into English using a consensus among three native English speakers (see Appendix A for details). This was done in order to verify that using two different languages did not alter the GPA results, as the consensus profile calculation is supposed to be done independently from the semantic information provided by the terminologies chosen by the observers.

### 2.5. Quantitative Behavioral Assessment

The behaviors shown by the 18 elephants in the 15 video clips were analyzed quantitatively, by a single observer using the continuous focal animal sampling technique, as defined by Martin and Bateson [43], and using a dedicated Behavioral Observation Research Interactive Software, BORIS [44]. The working ethogram used in this study is based on previous research on the behavior mainly of African elephants [45,46,47,48,49,50], and adapted according to the behaviors observed in the videos (Table 2). Trunk movements were considered a category on their own, even if some of them have been categorized as stereotypies in Asian elephants [51].

For videos in which multiple elephants were registered, each animal was observed individually, recording each behavior performed. When animals showed *events* behaviors simultaneously with *states* (e.g., elephant walks and “ear flap”), both were recorded. *States* behaviors were set as mutually exclusive because (the start of) a new state was considered most likely to reflect an intervening variation in the animal’s motivation and emotional state. Then, the total time in which each animal was visible in the video was calculated, excluding the time in which they were out of sight. The relative duration of each behavior was calculated over the total visible time of that video. The frequencies have been expressed as the number of occurrences of the behavior on the total time of the video.

### 2.6. Relationship between Quantitative and Qualitative Data

Spearman rank correlation analyses were performed to correlate scores on the first and second PCA dimension axis, separately calculated for children, adults, and experts’ groups with the elephants’ different behavioral patterns. The behaviors shown overall one or two times in the 15 videos were not included in the analysis, while others were grouped. Therefore, the behaviors included in the statistical analysis were: walk, walk backward, stand, feeding (expressed as the sum of eating behavior and feeding by tourists), ear open, ear flap, trunk swirling, trunk movements, trunk manipulation, and rocking. 

Statistical analyses were carried out using IBM SPSS Statistics 21.0 (SPSS Inc., Chicago, IL, USA) and Excel for Windows 2007 (Microsoft Inc., Redmond, WA, USA). 

## 3. Results

### 3.1. Observer Plot and Their Statistical Significance (Consensus Profile)

Observers generated a total of 641 terms to describe the elephants they were shown, with an average of 17.32 terms (range 5 to 39) per observer. In particular, the children group came up with an average of 11.57 terms (min: 5, max: 17); the adult group an average of 23.07 terms (min: 12, max: 39); and the expert group an average of 17.9 terms (min: 9, max: 28).

The Procrustes statistic values of the three consensus profiles (adults, children, and experts) are presented in Table 3, both as resulting from the Italian and from the English version of the terms. Overall, as expected, the results concerning the two languages were quite similar. Therefore, as use of English terms appears to be the usual procedure in the literature (e.g., [40]), only the results regarding the English will be presented and discussed further in the present paper.

The GPA showed that the consensus profile explained a significantly higher percentage of the variation between observer matrices than the mean of 100 randomized profiles, meaning that none of the consensus profiles was an artefact of GPA procedures. 

**Figure 1:**
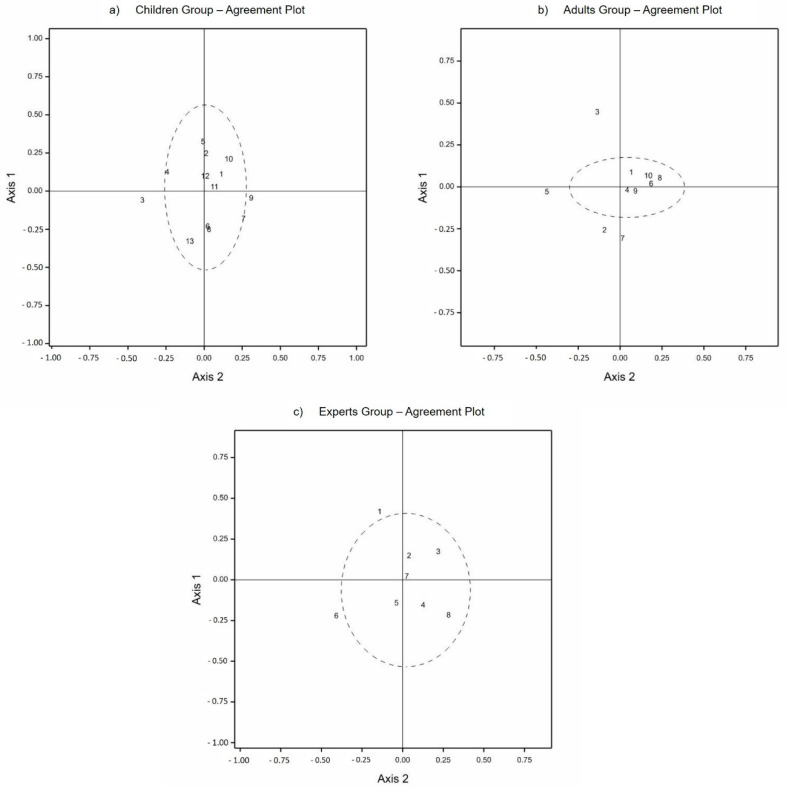
Observer plots of (**a**) children, (**b**) adults, and (**c**) experts.

The observer plots, after the outlier observer reduction, are shown in Figure 1. These plots reflect the relative distance between individual observers as a measure of the level of consensus between individual observer assessment. Numbers represent individual observers, while the dotted circles enclose a 95% confidence region for what may be considered the normal population of observers.

### 3.2. Interpretation of the Consensus Profile

The first dimension of the children’s consensus profile explains 27.1% of the variation between the elephants’ emotional state in the 15 videos, while the second dimension explains 19.4% of this variation. The first dimension of the adult consensus profile explains 23.7% of the variation, while the second dimension explains 15.8%. Finally, the first dimension of the expert consensus profile explains 21.8% of the variation, while the second one explains 17% of this variation.

To give a more general overview of the observer interpretations, Table 4 lists the two terms which held the highest positive and negative correlations with dimensions 1 and 2 for each observer, divided by groups.

In particular, the terms used most frequently by the 13 children to characterize the first dimension of the consensus profile were ‘free’, ‘friendly’, ‘hungry’, and ‘playful’ versus ‘sad’, ‘bored’, ‘apathetic’, and ‘stressed’. The terms used most frequently to characterize the second dimension of the consensus profile were ‘bored’, ‘sleepy’, ‘tired’, and ‘apathetic’ versus ‘friendly’, ‘hungry’, ‘sad’, and ‘curious’.

Moreover, the terms used most frequently by the 10 adults to characterize the first dimension of the consensus profile were ‘united’ and ‘gregarious’ versus ‘annoyed’ and ‘apathetic’, while the terms used most frequently to characterize the second dimension of the consensus profile were ‘agitated’, and ‘confident’ versus ‘angry’ and ‘bored’. However, even if adult observers mostly used different descriptors, their meaning was similar (e.g., ‘united’ and ‘gregarious’) or, in any case, coherent with different nuances of the same behavior and thus provided a comprehensive characterization of it (e.g., ‘hurried’, ‘restless’).

Finally, the terms used most frequently by the eight experts to characterize the first dimension of the consensus profile were ‘active’ and ‘excited’ versus ‘agitated’ and ‘bored’, while the terms used most frequently to characterize the second dimension of the consensus profile were ‘agitated’ and ‘annoyed’ versus ‘calm’ and ‘relaxed’. Nevertheless, in general, where observers used different terms, the meanings of these terms tended to be either similar in mood/tone (e.g., ‘agitated/jumpy/wary’ and ‘bored/boredom’) or complement each other in mood/tone (e.g., ‘in alert/on the defensive, ‘group cohesion/integrated between them’). In some cases, however, terms appear to contradict each other in tone (e.g., ‘excited’ and ‘quiet’, or ‘safe’ and ‘wary’ and ‘tension’).

**Figure 2:**
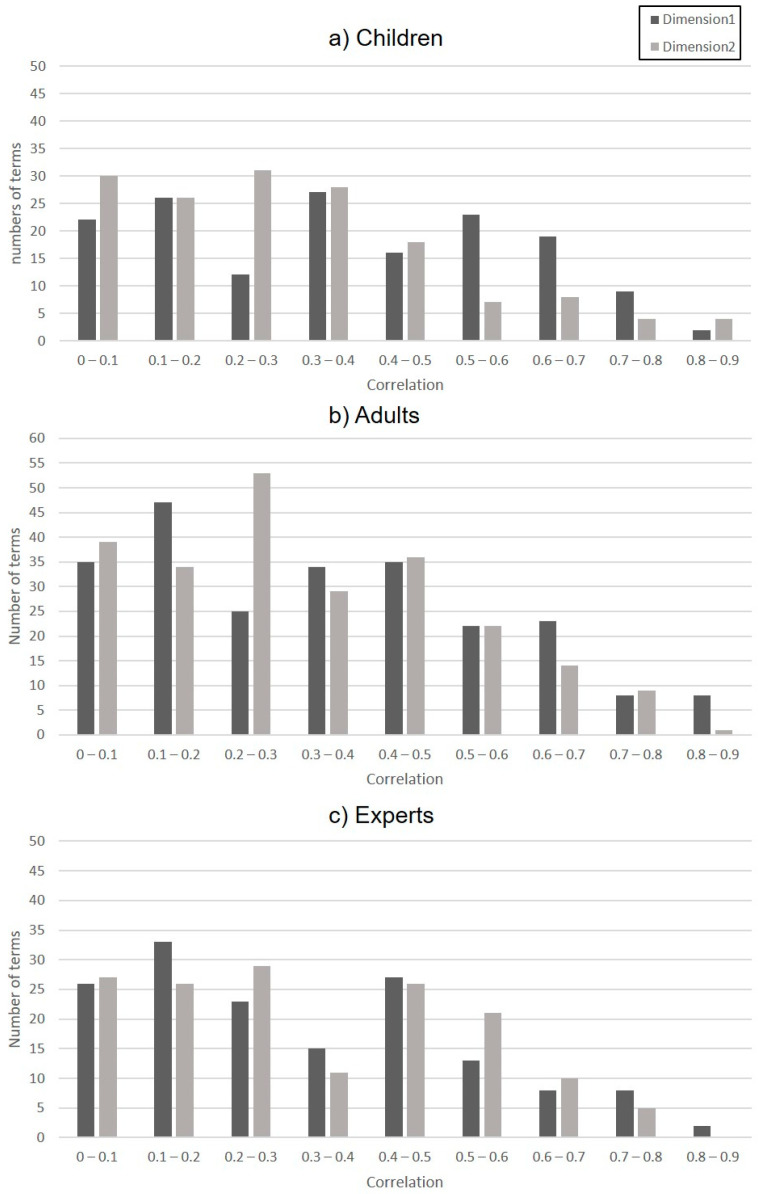
Correlation of (**a**) children, (**b**) adults, and (**c**) expert observer terms with dimension 1 and dimension 2.

Figure 2 indicates the strength of correlation (r-values) for all descriptors of dimension 1 and dimension 2. All the descriptors used by the three observer groups show a weak level of correlation on both dimensions.

### 3.3. Scatter Plots

**Figure 3:**
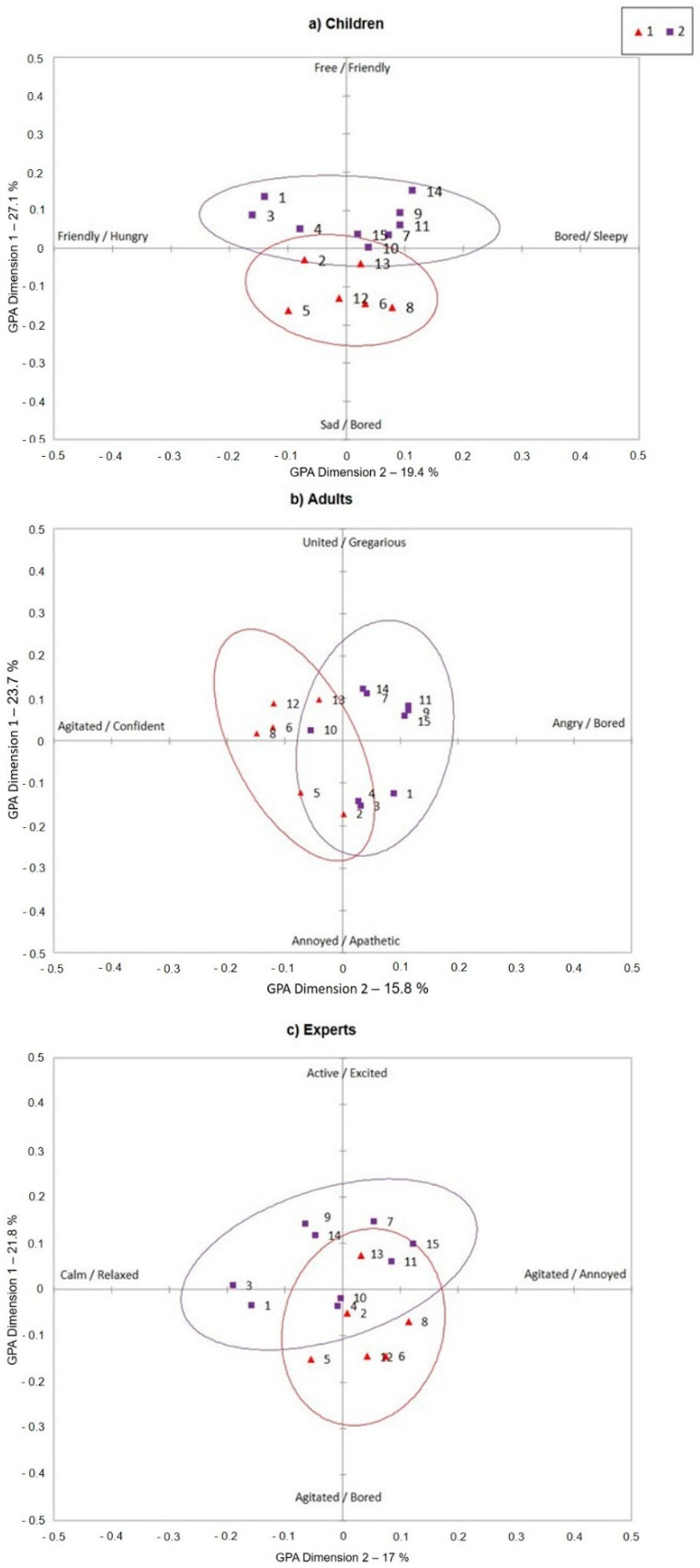
Generalized Procrustes Analysis (GPA) consensus score—elephant video scatter plots. The scatter plots represent the distribution of the videos (represented by the 1–15 numbers) on dimensions 1 and 2 of the consensus profile for (**a**) children, (**b**) adults, and (**c**) experts and the separation between videos representing captive (red triangles) and semi-captive (purple squares).

The videos were subsequently classified according to their management typology (captive or semi-captive—see Materials and Methods for definition). Videos representing animals in captivity were assigned the number 1, while videos representing animals in semi-captivity were assigned the number 2, accordingly with Table 1. Therefore, three elephant scatter plots (Figure 3) were created, one for each observer group, based on the consensus score of each observer. The plots describe how the animals shown in the 15 videos distribute along the two main dimensions according to observer’s perception.

The animals, represented by the videos, are evenly distributed over the two dimensions in children, adults, and experts’ plots, which suggests that these dimensions adequately characterize observed variances in behavioral expression.

The scatter plot obtained from the children’ consensus score (Figure 3a) shows a clear separation between the captive and semi-captive elephant group. The captive elephants represented by the red triangles are shifted towards the negative side of the first dimension (sad/bored as opposed to free/friendly) and equally distributed between the positive and negative side of the second dimension (bored/sleepy and friendly/hungry). The elephants in semi-captivity, represented by the purple squares, are shifted towards the positive side of the first dimension (free/friendly as opposed to sad/bored) and positive side of the second dimension (bored/sleepy as opposed to friendly/hungry). 

The scatter plot obtained from the adults’ consensus score (Figure 3b) also shows a clear separation between the captive and semi-captive elephant group. The captive elephants are shifted towards the positive side of the first dimension (united/gregarious as opposed to annoyed/apathetic) and the negative side of the second dimension (angry/bored as opposed to agitated/confident). The elephants in semi-captivity are shifted towards the positive side of the first dimension (united/gregarious as opposed to annoyed/apathetic) and positive side of the second dimension (agitated/confident as opposed to angry/bored).

Finally, the scatter plot obtained from the experts’ consensus score (Figure 3c) shows that the captive elephants are shifted towards the negative side of the first dimension (agitated/bored as opposed to active/excited) and the positive side of the second dimension (agitated/annoyed as opposed to calm/relaxed), while the elephants in semi-captivity are shifted towards the positive side of the first dimension (active/excited as opposed to agitated/bored) and negative side of the second dimension (calm/relaxed as opposed to agitated/annoyed).

Interestingly, videos 2 and 13, showing captive elephants and video 10, showing semi-captive elephants are included in the overlap area in all the three observers’ groups. 

### 3.4. The Correlation between Quantitative and Qualitative Data 

Table 5 presents the results of the Spearman correlation, used to investigate the relationship between the behaviors recorded on the videos through the quantitative evaluation and the projection of each video on the first and second dimensions obtained with the GPA. The table reports the moderate and weak correlation (r_s_ > ±0.5) and a statistical level of α ≤ 0.01.

The scores of the first dimension of the children group negatively correlate with elephants standing still (r_s_ = −0.64, *n* = 15, *p* = 0.01), and showing “trunk swirling” behavior (r_s_ = −0.70, *n* = 15, *p* < 0.01). Those results indicate that the elephants described as being more free/friendly on GPA dimension 1 (as opposed to sadder/more bored) spent a smaller proportion of time standing still and had fewer trunk swirling events. The scores of the second dimension negatively correlate with the “feeding” behavior (r_s_ = −0.75, *n* = 15, *p* = 0.01) and positively with the proportion of time spent walking (r_s_ = 0.68, *n* = 15, *p* < 0.01), indicating that elephants described as more friendly/hungry on GPA dimension 2 (as opposed to more bored/sleepy) spent a higher proportion of time eating and spent a smaller proportion of time walking.

The scores of the first dimension of the adults group positively correlate with elephants walking (r_s_ = 0.82, *n* = 15, *p* < 0.01), indicating that elephants described as being more united/gregarious on GPA dimension 1 (as opposed to more annoyed/apathetic) spent a larger proportion of time walking. The scores of the second dimension correlated negatively with “trunk swirling” (r_s_ = −0.77, *n* = 15, *p* < 0.01), indicating that elephants described as being angrier/more bored on GPA dimension 2 (as opposed to more agitated/confident) had more trunk swirling events.

Finally, the scores of the first dimension of the experts group positively correlate with elephants walking (r_s_ = 0.74, *n* = 15, *p* < 0.01) and negatively with “trunk swirling” (r_s_ = −0.73, *n* = 15, *p* < 0.01). Therefore, elephants described as being more active/excited on GPA dimension 1 (as opposed to more agitated/bored) had fewer trunk swirling events and spent a larger proportion of time walking.

## 4. Discussion

As a part of a larger project aimed at developing a protocol to specifically assess the welfare of elephants kept in semi-captive environments and involved in close-up AVIs [17], to be coupled with an overall ethical evaluation of the AVI themselves [52], the present study aimed to investigate how three groups of people, differing in age and knowledge of the species (i.e., children, non-expert adults, and experts), perceived the behavior of African elephants (*Loxodonta africana*) managed in captive and semi-captive environments, and whether the descriptors they generated were different for the elephants living under the two different management systems. Moreover, it also aimed to investigate the possible correlations between behavioral patterns observed and quantified using a traditional quantitative method applied in ethology and the results of the FCP. It is important to note that, in the context of the University of Padua’s project, the result obtained in the present study from the experts will be included in the protocol section regarding welfare assessment. Instead, results obtained by adults and children will be linked to the protocol section in which the AVI effect on the stakeholder “visitor” is assessed during the overall ethical assessment of AVIs. This notwithstanding, it is worth describing, in the context of the present paper, to what extent the results of the naïve observers’ group were similar to those of experts.

To our knowledge, the FCP and QBA methodologies were previously applied to African elephants just once, by Wemelsfelder and colleagues [53]. Twelve observers, of whom four were elephant experts and eight farm animal experts, assessed 28 clips taken at the Amboseli National Park, and 8 clips recorded in a UK Zoo/Safari park using a FCP methodology. Results showed good agreement between observers, who managed to come up with convergent terminologies, and a meaningful dimension relevant to health and welfare. From the word chart of two elephant expert observers presented in [53], it can be noted that the terms used by the elephant experts are similar to the terms used by the experts engaged in this study, although the language originally used by the respondents of the present study was not the same as that of respondents in the Wemelsfelder and colleagues study [53].

In the present study, all three observer groups, regardless of age and knowledge of the species, achieved good agreement in their qualitative assessment of the emotional expression of the African elephants. The consensus profiles of the children, adults, and expert groups explained 60%, 70%, and 68% of the variation, respectively. The statistical software is supposed to work independently from the semantic information provided by the terminologies chosen by the observers. However, it was created for terms generated in the English language. In this study, the descriptors gave by observers were in Italian, and then translated into English. By repeating the same analyses with both Italian and English terms, no differences in results were found. Therefore, it is unlikely that the different language originally used is the explanation of such variability. This notwithstanding, it would be interesting to assess whether different results could be obtained by involving English children, adults, and experts in the same study or asking Italian respondents to generate terms directly in English. Moreover, it would be interesting to perform the same study with native African observers living in close proximity to elephants, as familiarity with the animal species can affect QBA results [54]. Language notwithstanding, the terms used by the experts in the present study were similar to the list of terms included in protocols already developed to assess the welfare of zoo elephants [14,16]. From the 12 terms included in those protocols, we can note that terms such as “content”, “relaxed”, “agitated”, “tense”, “frustrated”, “wary”, “playful” appear in the results, defining positively and negatively the first two dimensions. Although this finding could be due to experts being aware of such protocols, it is interesting to note that all the three groups in the present study used a somewhat similar vocabulary to describe the elephant’s emotional state. For example, the negative end of the first axis was characterized by words such as “stressed”, “apathetic”, “bored” for children, “annoyed”, “sense of disappointment”, “apathetic”, “repetitive” for adults and “frustrated”, “bored/boredom”, “stereotyped” for experts. The aforementioned finding suggests that children and naïve adults, in the recreational moments of close-up experiences with the animals, can have a rough perception of the general emotional state of the animals with which they are interacting, and thus, indirectly of their welfare.

In agreement with previous studies, this methodology discriminated between animals held in two different types of management: captive and semi-captive. For example, Temple and colleagues [55], assessing the Iberian pig welfare through the QBA, found that this methodology was useful to discriminate farms (intensive or extensive rearing conditions) on the basis of the expression of behavior. From the distribution of the elephant videos on the two main dimensions of the GPA, it can be seen that, although there is a slight overlap, there is a separation between the elephants held in the captive or semi-captive management typology for all three groups of observers, with the animals in semi-activity moved towards the free/friendly or active/excited end. In contrast, the animals in captivity moved towards angry/bored.

This finding suggests that using adjectives generated exclusively in a zoological context when developing a QBA for elephants in semi-captive conditions may increase the risk of missing some welfare relevant points specific to the semi-captive context. The inclusion in the present study of distinct facilities, with varying typologies of management, allowed to create a “baseline” list of adjectives as vast as possible that could include most of the emotions expressed by the animals both in the zoo context and in contexts that could somewhat differ from the zoo one. It is interesting to note that, in the present study, naive observers were also able to discriminate between the two conditions, creating scatterplots in which there was an even greater degree of separation than amongst experts.

This finding agrees with the findings of Duijvesteijn and colleagues [33] and Wemelsfelder and colleagues [41], highlighting the importance of involving participants from different backgrounds and with a varying degree of familiarity with the studied animal species in order to obtain a balanced assessment of animal welfare from a QBA study [28,33,41]. Breeders or people who work with animals on a daily basis, were found to assess animal welfare more positively, focusing more on health than other stakeholder groups, while urban citizens or animal scientists were found to perceive natural behavior as the most important feature [33,41,56,57]. However, this should not lead to an underestimation of the importance of knowledge of the species, and of expertise in animal welfare assessment, as pitfalls could arise when people with little or no experience have to evaluate welfare without expert guidance.

Since zoos and facilities that offer “elephant experiences” to visitors usually rely on the sale of tickets to sustain themselves, they should both manage their animals according to objectively assessed good welfare levels and understand the factors that can affect the visitors’ perception of the welfare of the animals. This is significant since visitors are likely to choose facilities according to their perceptions of the emotional states of the animals in those facilities. Based on findings from the present study, elephants housed in less restrictive management environments were scored by naïve adults and children (i.e., average potential tourists) as tending to more positive emotional states.

Moreover, since education is one reason for involving animals in AVIs, it is important to promote both their welfare and the expression of their species-specific natural behavior. It has been shown that seeing animals express their natural behavior enhances the emotional value of observing them and this, in turn, increase visitors’ conservation-mindedness [58].

The present study also highlighted the potential benefits of involving children when assessing the perception of the emotional state of animals in controlled environments (e.g., high ability to discriminate between management conditions), as well as the limits of such an approach (e.g., low agreement in their use and scoring of terms). Children represent not only the main users of animal facilities but also the new generation on whose sensitivity conservation of biodiversity is likely to depend in the future. As far as we know, this study represents the first time that a group of children is included in a qualitative behavioural assessment, potentially opening a new path in exploring children’s perception of emotions in non-human species. 

The present study found correspondence between FCP results and quantitative assessment of behavior in the same way found by Rousing and Wemelsfelder [23], and Rutherford and colleagues [24].“Trunk swirling” was associated with the negative end of the first axis (“bored”) for experts, supporting the idea of this behavior as a possible correlate for mainly negative emotional states in elephants. In the scientific literature, “trunk swirling” has been described as a stereotypy for Asian elephants by de Mel and colleagues [23], and the present finding further supports its possible association with mainly negative mental states, at least in the perception of observers. However, the link between stereotypies and compromised welfare is a complex one, as, for example, stereotypies can emancipate from their causal situation, although, where data exist, in the 68% of cases, the situations which cause/increase them also decrease welfare [59]. Moreover, as a general rule, a single behavior is better evaluated in the context of the other behaviors and postures contextually shown by the animal expressing it. Of course, the correlation goes both ways and it may be possible that experts generated more negative adjectives because they saw more stereotypic behavior and recognized it for what it was. However, there were some similarities among the three groups in this respect, too. “Trunk swirling” was associated to the negative end of the first axis (“sad”, “stressed”) for children, and the negative end of the second axis for adults (“frustrated”). Such findings suggest that animals performing such behavior are perceived even by naïve observers as being in a more negative emotional state than those not doing so. Alternatively, performing such behavior could be associated with other aspects of the elephants’ demeanor that are interpreted as being associated with negative emotional states in the perception of people, irrespectively of their expertise with the species. Other correlations can be found for “walking” and “feeding” behavior. The results showed that adults described elephants that spent a higher proportion of time walking as more united/gregarious and the experts as more active/excited. However, in contrast, the children described the elephants as more bored/sleepy when the elephants spent a higher proportion of time walking. The children group also described the elephants that spent a higher proportion of time eating as more friendly/hungry on the second axis.

Using a FCP methodology, three groups of people (i.e., children, non-expert adults, and experts) assessed video-recordings of African elephants (*Loxodonta africana*) managed in captive and semi-captive environments, coming up with a similar terminology to that used by experts in other studies, both in zoos elephants [14,16], and in elephants living in more natural conditions [53]. Moreover, they were also able to differentiate, to some extent, between videos recordings of elephants in captive environments, and those in semi-captive environments, generally associating more emotionally positive descriptors to the latter. Although the present study did not aim at ranking different forms of management in terms of welfare, it is important to note that videos from semi-captive management projected somewhat differently from those in captive management in the experts’ consensus, suggesting that the terms generated in the two conditions either differed in themselves or their scores did (or possibly both). This suggests that the validity of using methods and protocols designed for captive conditions should not be taken for granted when assessing the welfare of semi captive individuals. 

It is suggested that the animals’ housing conditions could influence observer perception regarding the welfare of the animal, and consequently the QBA rating [60]. Depictions of the natural environment in videos of elephants under semi-captive management might create contextual bias among observers, leading them to score a more positive affective state in these situations. However, since Wemelsfelder et al. [61] did not detect serious distortions in observer assessment of pigs’ expressions in different contexts, the difference in scoring found in the present study is likely to be due more to the behavior of the elephants themselves, than to different visual contexts. Furthermore, an interesting correlation was found between a behavior (i.e., trunk swirling) described in the scientific literature as a stereotypy, and negative emotional descriptors in all the groups of observers. The finding that people can perceive an animal performing a stereotypy as “stressed”, “frustrated”, and/or “sad”, further highlights the importance of ensuring conditions conducive to good welfare and natural behavior in animals exposed to the public, not only because they are of outmost importance to the animals’ quality of life, but also for the impact they can have on members of the public and their conservation mindness. Although we cannot rule this out completely, it is unlikely that the differences found were due to the different periods in which the videos were filmed (video collection ranged from October to December). On one hand, there is evidence of seasonal changes in movement [62] and social structure amongst wild elephants, depending on rainfall patterns and the availability of resources. In fact, in cases of abundance of resources, elephant groups tend to stay together. In contrast, groups tend to split when competition for resources is high. Such behavior is typical of their fission–fusion social structure [63]. Moreover, different seasons might present a significant change of vegetation in certain areas, influencing the food intake behavior (grazing vs. browsing) [64]. On the other hand, to our knowledge, changes in behavior between seasons has not been described under captive conditions in African elephants, as resources are controlled and thus unlikely to affect behavior. In this regard, it is important to note that the only facility included in this study that was not in South Africa was a zoo where the elephants’ diet is controlled thorough the year. 

## 5. Conclusions

During the last decade, qualitative behavior assessment has been progressively validated and applied to a wide range of domestic species and some wild species. Recently, QBA has also been included in protocols to assess elephants’ welfare in zoos. In this study, three groups of observers were asked to evaluate their perception of the elephant’s demeanor using the FCP method. The African elephants included in the assessment were managed not only in captive but also semi-captive environments and, to our knowledge this is the first application of FCP to assess “elephants’ experiences” in semi-captive facilities. Results suggest that developing a QBA for elephants in semi-captive conditions by the use of adjectives generated exclusively in a zoological context may be a sub-optimal approach. In this regard, the descriptors generated in the present study could be a more suitable option in designing a QBA for welfare assessment of elephants both in zoos and in “elephants’ experiences” facilities. Given that zoos and interactions between zoo visitors and animals play a role in conservation education, naive observers’ perceptions of the emotional state of the animals could be a useful tool to assess their welfare. In this study, naïve observers, particularly children, deemed to be representative of zoo visitors and “elephants’ experiences” participants, for the first time were included in the QBA assessment. Due to the link between the emotional value of seeing wild animals in a controlled environment and conservation-mindedness [58], further research is required to investigate which factors influence the naïve people’s perception of animals’ demeanor. Finally, this study suggests that the captive and semi-captive environment in which the animals are kept may influence African elephant emotional expression. However, further studies are needed to investigate the impact of the environment (captive and semi-captive) on African elephants’ demeanor.

## Figures and Tables

**Table 1:** Summary of each study location, including the elephants’ name, their age at the study time, the presence or absence of a training program, whether the animals were involved in interactions with visitors, and whether held in the captive or semi-captive management typology.

Institution	Elephants Included in the Study	Age Range (Years Old)	Training	Interaction with Visitors	Management Typology
**Zoo Safari Ravenna**	Dumbo (female), Robin (female)	35–45	No	Yes	Captive
**Indalu Game Reserve**	Mooketsi (male), Bakari (male), Teboco (male), Amari (female), Madiwa (female), Shanti (female)	11–23	Yes	Yes	Semi-captive
**Garden Route Game Lodge**	Salati (male), Moya (male)	17–35	No	No	Semi-captive
**Adventures with Elephants**	Chishuru (male), Chova (male), Nuanedi (female), Shan (female), Mussina (female)	15–20	Yes	Yes	Semi-captive
**National Zoological Garden**	Charlie (male), Thandi (female), Landa (female)	33–36	No	No	Captive

**Table 2:** Working ethogram. Behaviors marked with “§” represent *events*.

Category	Behavior	Definition
Locomotion	Walk	Animal takes more than 2 steps forward, but not in a stereotypic pattern.
	Walk backwards	Animal takes more than 2 steps backwards, but not in a stereotypic pattern.
	Change direction §	Animal makes a change in its direction while walking.
Standing	Stand	Animal stands in a still position with its eyes open or closed, possibly exhibiting other behaviors at the same time but without changing place.
Feeding	Eat	Animal is in the process of putting food in its mouth with its trunk.
	Foraging	Animal is actively searching for food with its trunk.
Eliminatory	Urinate	Animal discharges urine out of the body.
	Defecate	Animal discharges feces out of the body.
Ear Movements	Ear open	Animal keeps one or both ears separate from the body with an angle ≤ 90°.
	Ear flap §	Animal moves one or both ears from 0° to ≤90° and from ≤90° back to 0°.
Trunk movements	Trunk swirling §	Animal swirls downwards its trunk’s first half, second half or both on its circular axis [51].
	Trunk up §	Animal swirls its trunk as before but upwards.
	Trunk swing §	Animal moves its trunk from one side to the other (left <-> right; up <-> down) once or in a repetitive manner.
Self-directed movements of the trunk	Trunk—body part §	Animal touches its own body in specified part with its trunk.
Investigative movements of the trunk	Trunk forward §	Animal stretches its trunk toward something it wants to reach.
	Trunk manipulation	Animal picks up something in its trunk and physically interacts with it by pulling, pressing, rotating, or moving it in general.
Head movements	Head shake §	Animal heavily agitates its head.
Stereotypies	Pacing	Animal takes steps forward or backwards in an unvarying, repetitive manner. In this study, we are consider pacing after the repetitive fifth step forward/backwards.
	Rocking	Side-to-side and/or back-and-forth repetitive swaying of the body.
Out of sight	Out of sight	Animal is not visible, partially, or completely.
Commands	Commanded trunk up §	Animal performs a “trunk up” after being commanded to do so.
	Commanded change direction §	Animal changes direction after being commanded to do so.
	Commanded yes/no §	Animal moves its head in a nodding manner or from side to side after being commanded to do so.
Interactions with tourists	Being fed by tourists	Animal receives food in its mouth or in its trunk by a tourist.

**Table 3:** Procrustes statistics for the observer groups (*p* < 0.001).

Observer Group	Consensus Profile (%)		Randomized Procrustes Statistics (Mean ± SD)		Student *t*-Test (df = 99)	
	Eng.	Ita.	Eng.	Ita.	Eng.	Ita.
Children	57.20	60.37	53.29 ± 0.35	55.55 ± 0.42	55.3	57.3
Adults	70.37	70.37	66.24 ± 0.32	66.24 ± 0.32	67.9	67.9
Experts	68.50	68.50	65.10 ± 0.35	65.10 ± 0.35	66.8	66.8

**Table 4:** Terms used by the observer groups showing the highest correlations with the first and second dimensions of the consensus profile (axis 1 and axis 2). The loading values near 0 indicate no correlation, whereas if near 1 indicates highly and positively correlated values. The closer the values are to −1, the more inverse the correlation is. The values in brackets indicate the number of observers using the specific terms, unless used by 1 observer.

Group	Axis	Positive Correlation	Negative Correlation
Children			
Axis 1		Free (3), Friendly (2), Hungry (3), Playful (2), Amused, Himself/itself, Joyful Loadings varying from 0.35 to 0.77	Sad (6), Bored (2), Apathetic, Stressed, Impatient, Sleepy, Wants to go out Loadings varying from −0.19 to −0.89
Axis 2		Bored (3), Sleepy (2), Tired (2), Apathetic, Compact, Nervous, Patient, Scared, Stressed Loadings varying from 0.18 to 0.87	Friendly (3), Hungry (2), Sad (2), Curious (2), Calm, Feels bad, Free, Stuck Loadings varying from −0.28 to −0.88
Adults			
Axis 1		United (2), Gregarious, Hurried, Impatient, Jumpy, Ordered, Relieved, Restless, Supportive Loadings varying from 0.42 to 0.88	Annoyed, Apathetic, Confident, Greedy, Happy, Hungry, Repetitive, Skeptical, Sense of disappointment, Uninterested Loadings varying from −0.38 to −0.85
Axis 2		Agitated, Confident, Content, Curious, Free, Keeps distance, Lost, Methodical, Not friendly, Skeptical Loadings varying from 0.39 to 0.77	Angry, Bored, Frustrated, Happy, Impatient, Jumpy, Not happy to return, Stereotyped, Stressed, Waiting Loadings varying from −0.28 to −0.83
Experts			
Axis 1		Active, Excited, Group cohesion, Integrated between them, Naturalness, On the defensive, Quiet, Safety Loadings varying from 0.47 to 0.77	Agitated, Bored, Boredom, Friendly, Frustrated, Seeking attention, Stereotyped, Waiting Loading varying from −0.43 to −0.83
Axis 2		Agitated, Annoyed, Expectation, In alert, Jumpy, Safe, Tension, Wary Loadings varying from 0.43 to 0.70	Calm (2), Relaxed (2), Complicity, Curious, Obedient, Satisfied Loadings varying from −0.07 to −0.78

**Table 5:** Spearman’s rho correlations between generalized Procrustes analysis (GPA) scores on dimensions 1 and 2 of the three observer groups separately, and the relative duration/frequency § of the behavioral patterns shown by the 18 elephants in the 15 videos.

Group	Dimension	Spearman’s Rho (r_s_)	*p*-Value	Sample Size	Behavior
Children	First Dimension	−0.641	0.010	15	Stand
		−0.704	0.003	15	Trunk swirling §
	Second Dimension	0.686	0.005	15	Walk
		−0.753	0.001	15	Feeding
Adults	First Dimension	0.829	0.000	15	Walk
	Second Dimension	−0.777	0.001	15	Trunk swirling §
Experts	First Dimension	0.742	0.002	15	Walk
		−0.733	0.002	15	Trunk swirling §

## Data Availability

The data presented in this study are available on request from the corresponding author.

## Appendix A

The observers gave their terms in Italian, which were then translated into English to verify that using two different languages did not alter the GPA results. The consensus profile calculation, in fact, is supposed to be done independently from the semantic information provided by the terminologies chosen by the observers.

Therefore, three different people were involved in the translation process (one native American speaker and two bilingual English-Italian speakers). The translators were given all the words used by the observers and asked to translate them independently.

Subsequently, the English translations were compared and checked for concordance between the three. If a concordance was found between two or three of the three translators, the word given by the majority was chosen. If the three people gave different translations of the terms, online translators were used (DeepL—Wordreference—Google Translate) to identify the best interpretation of the Italian word.

In some cases, different terms in Italian, which have similar meaning, were translated into the same term in English as a result of the previously described process (e.g., “appagato” and “soddisatto” were both translated into satisfied). In this case, if the two original Italian adjectives were used only by different observers, no action was taken, and both remained translated into the same English term, whereas if the same observer had used the two (or more) Italian terms that resulted in having been translated into the same English adjective, other correspondences were found so that each of the different Italian adjectives was translated into a different English one. In the example above, as one observer had used both adjectives to describe elephants, “appagato” was translated into fulfilled, while “soddisfatto” remained satisfied.
